# Trajectory Prediction-Enabled Self-Decision-Making for Autonomous Cleaning Robots in Semi-Structured Dynamic Campus Environments

**DOI:** 10.3390/s26072258

**Published:** 2026-04-06

**Authors:** Jie Peng, Zhengze Zhu, Qingsong Fan, Ranfei Xia, Zheng Yin

**Affiliations:** 1 School of Intelligent Connected Vehicle, Hubei University of Automotive Technology, Shiyan 442002, China; 202311315@huat.edu.cn; 2 Dongfeng Commercial Vehicle Co., Ltd., Shiyan 442001, China; xiarf@dfcv.com.cn (R.X.); yinzheng@dfcv.com.cn (Z.Y.); 3 International Joint Research Center of Automotive Cloud Computing and Simulation Control, Hubei University of Automotive Technology, Shiyan 442002, China; 4 Hubei Provincial Engineering (Technology) Research Center of Automotive Intelligent Networking and Electronic Control, Hubei University of Automotive Technology, Shiyan 442002, China; 5 Shiyan Key Laboratory of Air-Ground Crowd Cooperation Technology and Application, Hubei University of Automotive Technology, Shiyan 442002, China; 6 State Key Laboratory of Intelligent Manufacturing Equipment and Technology, Huazhong University of Science and Technology, Wuhan 430074, China; 7 School of Computer Science and Artificial Intelligence, Wuhan University of Technology, Wuhan 430070, China

**Keywords:** autonomous cleaning robots, self-decision-making, trajectory prediction

## Abstract

Autonomous cleaning robots operating in semi-structured dynamic environments must execute task-oriented motions while safely interacting with surrounding agents. These agents include pedestrians, vehicles, and other robots. In such environments (e.g., interaction-rich campus environments), reliable self-decision-making requires anticipating the future motions of surrounding agents rather than relying solely on reactive obstacle avoidance. This paper presents a trajectory prediction-enabled self-decision-making framework for autonomous cleaning robots in campus environments. A learning-based multi-agent trajectory prediction model is trained offline using public benchmarks and real-world operational data to capture typical interaction patterns in corridor-following, edge-cleaning, and intersection scenarios. The predicted trajectories are then incorporated as forward-looking priors into the robot’s online decision-making and planning process, enabling prediction-aware yielding, detouring, and task continuation decisions. The proposed framework is evaluated using real-world data-driven scenario reconstruction on a high-fidelity simulation platform that incorporates realistic vehicle dynamics and heterogeneous traffic participants. This evaluation focuses on short-horizon prediction performance and its impact on downstream decision-making stability. The results show that integrating trajectory prediction into the decision-making loop leads to more stable motion behavior and fewer abrupt adjustments in interaction scenarios. Under short-term prediction horizons, the evaluation results show that the proposed model achieves ADERate and FDERate exceeding 90% under predefined error thresholds, while lane-change prediction accuracy remains around 79%. In addition, the robot maintains stable speed tracking with only minor fluctuations under medium-density traffic conditions.

## 1. Introduction

Sanitation services have been under increasing pressure in recent years, largely due to workforce aging and limited recruitment among younger generations. In response, municipalities and campus administrations have begun introducing autonomous cleaning vehicles as a long-term operational alternative. Cost analyses in several cities suggest that continuous operation can partially offset initial deployment expenses, making these systems economically viable over time. Meanwhile, smart-city and low-carbon initiatives have further accelerated pilot deployments in campus and industrial park settings [1]. Unlike indoor service robots, most real-world deployments involve medium- to large-sized cleaning vehicles operating outdoors at relatively higher speeds [2,3]. These platforms are required to perform area-wide coverage tasks while complying with safety regulations and interacting with pedestrians, service vehicles, and other mobile robots. In practice, debris types and traffic patterns vary throughout the day, leading to operating conditions that are dynamic and only partially predictable. As a result, cleaning robots must balance route adherence with situational adaptation, especially in shared environments where human motion is irregular. Although the SAE automation levels are originally defined for on-road motor vehicles, the autonomy capability of the cleaning robots considered in this work can be regarded as comparable to SAE Level 4 automation within a constrained operational design domain (ODD), namely campus environments. Within this domain, the robot is expected to perform perception, prediction, decision-making, and motion planning autonomously while safely interacting with pedestrians and other mobile agents.

While many techniques developed for autonomous driving can be adapted to service robots, campus cleaning robots differ from conventional autonomous vehicles in several aspects. In particular, cleaning robots operate at low speeds in pedestrian-dominated environments and perform coverage-oriented service tasks rather than point-to-point transportation. These characteristics lead to different interaction patterns and decision-making requirements compared with road vehicles. A recurring difficulty in these environments is that purely reactive strategies are often insufficient. Obstacle-avoidance approaches based solely on instantaneous perception may perform adequately in sparse settings, but they tend to produce conservative maneuvers or oscillatory behaviors in narrow corridors or crowded walkways. Moreover, cleaning tasks typically involve boundary-following and coverage constraints, where unnecessary stops or detours reduce operational efficiency [4,5]. These observations further indicate that decision-making mechanisms should incorporate short-term anticipation of surrounding agents’ motion, rather than relying exclusively on reactive responses.

Existing studies on Mobile Robot (MR) navigation and autonomous driving have explored a wide range of decision-making and planning strategies, from rule-based and optimization-driven methods to learning-based approaches [6]. In parallel, recent advances in trajectory prediction have demonstrated strong capability in modeling short-term future motions of pedestrians and vehicles using data-driven techniques [7]. However, in most practical systems, trajectory prediction and decision-making are treated as loosely coupled modules: prediction outputs are often used for risk assessment or evaluation, while downstream decisions remain primarily driven by current-state information [8,9]. As a result, the potential of trajectory prediction to directly inform task-level decision-making and interaction reasoning has not been fully exploited, particularly for autonomous service robots operating in semi-structured campus environments.

Motivated by these observations, this paper proposes a prediction-enabled self-decision-making framework for autonomous cleaning robots operating in dynamic campus environments. Instead of using trajectory prediction merely as an auxiliary perception output, predicted future motions of surrounding agents are explicitly incorporated into the decision-making loop as forward-looking priors. By conditioning decision evaluation and behavior selection on short-horizon multi-agent trajectory predictions, the proposed framework enables the robot to anticipate potential interactions and make more stable and task-consistent decisions. The focus of this work is not on pursuing highly complex long-horizon prediction models, but on designing a practical integration strategy that bridges learning-based trajectory prediction and task-oriented decision-making in a systematic manner.

The main contributions of this paper are summarized as follows:

- A prediction-enabled self-decision-making framework is proposed for autonomous cleaning robots operating in dynamic campus environments, where trajectory prediction is explicitly integrated into task-level decision-making rather than reactive collision avoidance.
- A learning-based multi-modal trajectory prediction module is incorporated as a forward-looking prior to support interaction-aware reasoning, allowing for the robot to account for multiple plausible future behaviors of surrounding agents.
- A high-fidelity simulation-based evaluation methodology is developed, where real operational data are replayed and reconstructed to systematically assess both trajectory prediction performance and decision robustness under diverse interaction conditions. Experimental results indicate that reliable short-horizon prediction is sufficient to significantly enhance decision quality, providing practical insights for real-world deployment.

The remainder of this paper is organized as follows: Section 2 reviews related work on mobile robot decision-making and trajectory prediction. Section 3 formulates the problem and presents an overview of the proposed system architecture. Section 4 details the trajectory prediction model and its integration into the self-decision-making framework. Section 5 reports experimental results based on high-fidelity simulation and real-data-driven scenario reconstruction. Finally, Section 6 concludes the paper and discusses directions for future work.

## 2. Related Works

### 2.1. Decision-Making and Navigation for Mobile Robots

Decision-making and navigation have long been central topics in mobile robotics and autonomous driving research. Classical approaches typically adopt a hierarchical and modular architecture, where high-level decision-making generates behavioral intentions and low-level planners compute feasible trajectories under kinematic and dynamic constraints. Early optimization-based trajectory generation methods, such as the Frenét-frame formulation proposed by Werling et al., enable smooth and dynamically feasible motion by decoupling longitudinal and lateral planning and optimizing jerk-based cost functions [10]. These methods provide strong motion feasibility guarantees and have been widely adopted in structured environments.

To improve robustness in complex and interactive scenarios, planning-oriented decision-making frameworks have been proposed to tightly couple decision and trajectory generation. Hu et al. emphasized the importance of planning-oriented autonomous driving, where decision-making explicitly considers downstream planning feasibility and interaction constraints [11]. Similarly, Ding et al. introduced spatio-temporal semantic corridors and later the EPSILON framework to enable efficient and interaction-aware planning in dense traffic environments [12,13]. These approaches demonstrate that incorporating semantic context and temporal consistency can significantly improve safety and efficiency, but they still rely heavily on manually designed prediction and cost models.

Uncertainty-aware decision-making has also received increasing attention. Zhang et al. proposed an efficient uncertainty-aware decision-making framework using guided branching to address stochastic behaviors and perception uncertainty in dense traffic [14]. By leveraging domain knowledge and semantic-level policies, such approaches balance computational efficiency and decision robustness, yet their performance strongly depends on the quality of behavior prediction and uncertainty modeling.

In parallel, learning-based decision-making has emerged as a powerful alternative. End-to-end learning approaches directly map sensory inputs to control commands, as demonstrated by Bojarski et al., showing the potential of data-driven decision pipelines [15]. Reinforcement learning (RL), particularly deep RL, further enables agents to learn complex decision policies through interaction with the environment. Distributional reinforcement learning methods, such as the distributional soft actor–critic (DSAC), explicitly model return uncertainty and have been successfully applied to autonomous driving decision-making in interactive scenarios [16,17,18]. These methods offer improved robustness and adaptability but often face challenges in interpretability and safety assurance.

Overall, existing decision-making and navigation methods span from optimization-based planners to learning-driven frameworks. While classical approaches provide interpretability and safety guarantees, learning-based methods offer superior adaptability. However, most prior work focuses on road vehicles, whereas campus cleaning robots operate in more confined spaces with frequent pedestrian interactions, motivating prediction-enhanced decision-making tailored to such environments.

### 2.2. Trajectory Prediction in Dynamic Environments

Trajectory prediction plays a crucial role in enabling proactive and safe decision-making in dynamic environments. Traditional prediction methods often assume simple motion models or rely on short-horizon extrapolation, which are insufficient in highly interactive scenarios. With the rise of data-driven techniques, learning-based trajectory prediction has become the dominant paradigm.

Early deep learning approaches rely on rasterized scene representations combined with convolutional neural networks. More recent methods adopt vectorized scene representations and attention-based architectures to better capture interactions among agents and map elements. The Transformer framework proposed by Vaswani et al. has played a central role in this transition and has since been adapted for motion forecasting tasks [19]. For example, Wayformer extends attention mechanisms to large-scale scenes, enabling the modeling of long-range dependencies while maintaining computational tractability [20]. Another line of research focuses on improving encoding efficiency in multi-agent settings. Zhou et al. proposed a query-centric prediction framework that separates scene representation from agent-specific coordinate systems, allowing for streaming scene encoding and parallel decoding across multiple agents [21]. This decoupling strategy reduces redundancy in agent-centric representations and facilitates real-time inference.

More recently, state-space and hybrid attention models have attracted attention for long-horizon forecasting. Huang et al. introduced Trajectory Mamba, combining selective state-space modeling with attention mechanisms to balance efficiency and expressiveness [22]. Compared with purely attention-based architectures, such hybrid models offer an alternative design trade-off between accuracy and computational cost. Beyond prediction accuracy alone, increasing emphasis has been placed on risk-aware and decision-oriented forecasting. Chen et al. developed the PODAR model to quantify collision risk by incorporating physical damage estimates and spatio-temporal uncertainty, thereby providing interpretable signals for downstream decision-making modules [23]. Hierarchical trajectory representations have also been explored to improve stability and interpretability in complex traffic scenarios [24]. Recent studies have also highlighted the growing role of human–robot interaction (HRI) in shared environments where robots must coexist with human operators [25]. Beyond purely motion-level interaction, cognitive-level communication and context understanding are increasingly important for improving transparency and collaboration between humans and robots [26]. Recent advances in large language models (LLMs) provide new opportunities for enabling semantic reasoning, task understanding, and natural-language interaction in robotic systems [27]. These capabilities may further enhance the interpretability and adaptability of autonomous decision-making frameworks in human-centered environments. Although promising, the integration of LLM-based reasoning into real-time motion planning remains an open challenge.

Despite these developments, most existing trajectory prediction models are evaluated on autonomous driving benchmarks. Campus environments, however, differ in several aspects, including dense pedestrian movement, irregular motion behaviors, and frequent low-speed interactions. These characteristics suggest that prediction models for service robots should prioritize not only accuracy but also computational efficiency and tight integration with task-level decision-making.

### 2.3. Summary and Motivation

Research on mobile robot navigation and decision-making has achieved reliable performance in structured or moderately dynamic settings. In such environments, reactive obstacle avoidance and rule-based planning remain practical solutions due to their simplicity and predictable computational cost. Difficulties arise, however, when robots operate in interaction-rich scenarios, where the future motion of surrounding agents directly influences the quality of decision-making. Although trajectory prediction methods have shown promising short-term forecasting capability for pedestrians and other mobile agents, prediction and behavior selection are still often implemented as loosely connected components.

In campus environments, autonomous cleaning robots must continuously satisfy coverage and boundary-following constraints while interacting with pedestrians, service vehicles, and other robots. Under these conditions, accurate prediction alone does not necessarily translate into stable or efficient robot behavior. The way prediction outputs are incorporated into the decision-making process becomes equally important.

This observation motivates the present study. Instead of pursuing increasingly complex prediction architectures, we investigate how prediction results can be structured and integrated within a practical decision-making framework. The emphasis is placed on system-level coordination between trajectory forecasting and task-oriented behavior selection, with the aim of supporting anticipatory and stable robot operation in dynamic campus environments.

## 3. Problem Definition and System Overview

### 3.1. Operational Scenarios

The cleaning robot considered in this work performs task-oriented navigation, typically following road boundaries or corridor edges to achieve consistent coverage. The operational area contains multiple orthogonal intersections and elongated corridor segments, where the robot frequently encounters pedestrians, other service robots, and occasionally slow-moving vehicles. Interactions are particularly common near intersections, where agents may approach from different directions, pause unpredictably, or abruptly change their intended motion.

**Figure 1:**
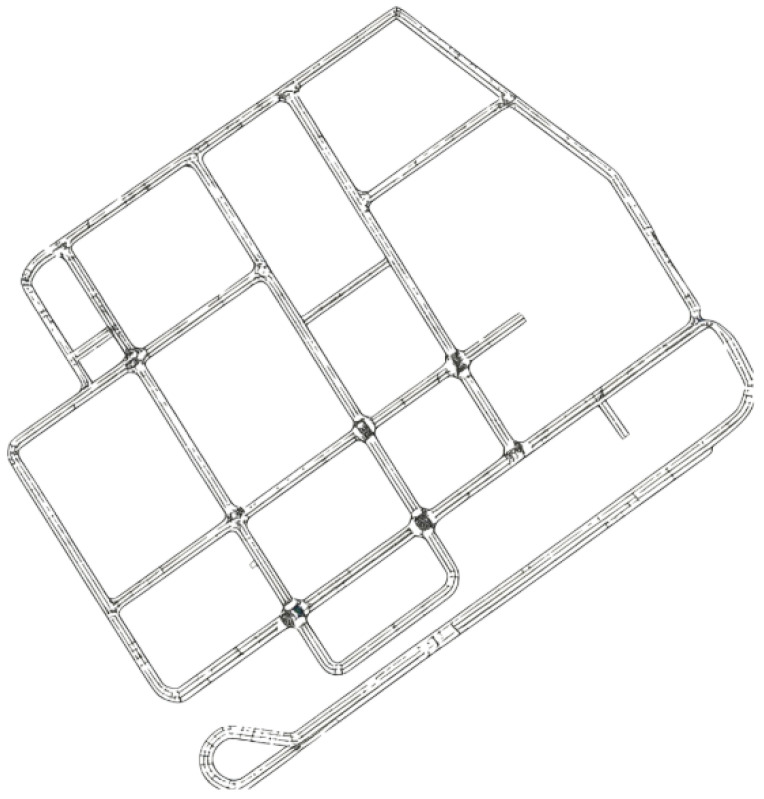
The semi-structured dynamic campus environments.

Figure 1 depicts the semi-structured campus environment used in our experiments. The road network forms a closed layout composed of corridors, junctions, and boundary-following segments. While the topological connectivity of the map remains fixed, the motion of surrounding agents varies over time and cannot be assumed deterministic. Such environments resemble typical campus or industrial park deployments in which cleaning robots are expected to operate continuously as part of routine maintenance. The evaluated operational scenarios include corridor traversal, intersection negotiation, and local detouring. During corridor following, the robot moves along narrow passages while maintaining a predefined lateral offset for edge-cleaning. Encounters with oncoming pedestrians or overtaking agents often require speed modulation or short yielding maneuvers. At multi-way junctions, the robot must estimate whether it is safe to proceed or whether deceleration is necessary before entering the intersection area. Temporary obstacles or interacting agents may also block the intended cleaning route, resulting in short deviations before task execution resumes.

In these situations, purely reactive navigation tends to produce frequent halts or oscillatory adjustments, especially under low-speed but interaction-dense conditions. Anticipating the short-term motion of surrounding agents can reduce such behavior fluctuations. For this reason, trajectory prediction is incorporated into the robot’s decision-making framework to support smoother and more task-consistent operation across different campus scenarios.

### 3.2. Problem Formulation

We consider the autonomous navigation and task execution problem of a cleaning robot operating in a campus environment with multiple dynamic agents. The robot is required to accomplish boundary-following and area-coverage tasks while safely interacting with pedestrians, other robots, and occasional vehicles. The environment is partially structured, defined by a fixed road topology and intersections, yet highly dynamic due to the stochastic behaviors of surrounding agents. At each discrete decision step *t*, the robot observes a state vector

(1) $$s_{t}=\left\{s_{t}^{ego},s_{t}^{env},s_{t}^{map}\right\}$$

where

- $s_{t}^{ego}$ represents the ego robot state, including position, velocity, heading, and recent control history;
- $s_{t}^{env}$ encodes the states of surrounding agents within a local perception range, represented by their recent motion histories (positions, velocities, headings, and agent types);
- $s_{t}^{map}$ contains local static environment information such as corridor geometry, intersection topology, and navigable boundaries.

This formulation follows a commonly used abstraction in data-driven decision-making systems.

Given the observed environment state, the robot employs a trajectory prediction module to estimate the future motion of surrounding agents over a finite horizon *H*:

(2) $${\hat{T}}_{t}=\left\{{\hat{\tau }}^{\left(i\right)}|i\in A\right\}$$

where each predicted trajectory

(3) $${\hat{\tau }}^{\left(i\right)}=\left\{{\hat{x}}_{t+1}^{\left(i\right)},\ldots ,{\hat{x}}_{t+H}^{\left(i\right)}\right\}$$

represents the anticipated future states of agent *i*.

The prediction horizon and accuracy requirements are designed to ensure lane-level and corridor-level decision reliability, enabling the robot to anticipate potential conflicts at intersections, narrow passages, and boundary-following segments. Rather than treating prediction as a standalone perception output, the predicted trajectories are explicitly incorporated as priors in the downstream decision-making process.

The robot’s decision-making problem is formulated as a constrained sequential decision process. At each time step, the robot selects an action $a_{t}\in A$, which corresponds to high-level motion commands (e.g., acceleration and steering or curvature-based controls). The decision policy $\pi (a_{t}|s_{t},{\hat{T}}_{t})$ is conditioned not only on the current state but also on the predicted future trajectories of surrounding agents. This enables the robot to evaluate candidate actions with respect to anticipated interactions rather than instantaneous observations alone.

The objective is to minimize a cumulative cost function over a finite horizon, where $H_{d}$ and $H_{p}$ denote the decision and prediction horizons, respectively,

(4) $$\min_{\pi }\;E\left[\sum_{k=0}^{H_{d}}\ell \left(s_{t+k},a_{t+k}\right)\right]$$

subject to dynamic feasibility and safety constraints derived from predicted agent motions. These constraints enforce collision avoidance, boundary compliance, and smooth motion execution.

To achieve real-time performance in dense interaction scenarios, the original constrained optimal control problem is transformed into a learning-based policy optimization problem, where the policy is trained offline and executed online with low computational overhead.

The effectiveness of the proposed decision-making framework is evaluated using multi-dimensional performance metrics that reflect realistic operational requirements. These include safety-related measures derived from predicted interaction risk, motion smoothness indicators, task efficiency, and rule compliance. Such metrics allow for quantitative assessment of how trajectory prediction contributes to safer and more stable decision-making behaviors in interaction-rich environments.

### 3.3. System Architecture

**Figure 2:**
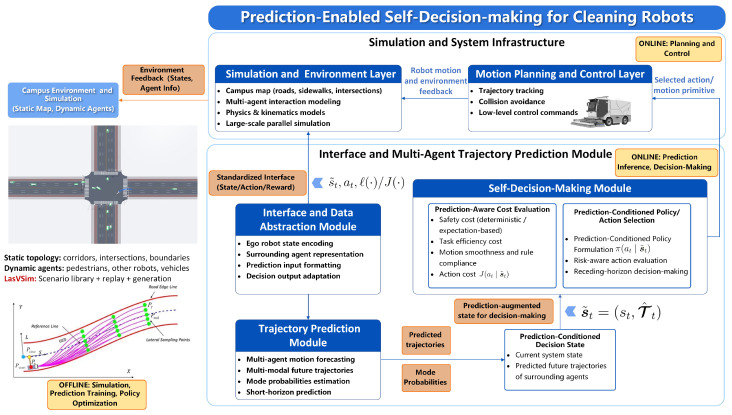
System architecture of the proposed prediction-enabled self-decision-making framework for autonomous cleaning robots in campus environments.

The overall architecture of the proposed prediction-enabled self-decision-making framework for autonomous cleaning robots in campus environments is illustrated in Figure 2. The system is organized as a closed-loop pipeline composed of five main components: the *Simulation and Environment Layer*, the *Interface and Data Abstraction Module*, the *Trajectory Prediction Module*, and the *Self-Decision-Making Module*, followed by the *Motion Planning and Control Layer*. The entire framework is implemented and evaluated on a large-scale simulation platform (i.e., LasVSim), which provides campus-level environment modeling and multi-agent interaction capabilities.

As shown in Figure 2, the *Simulation and Environment Layer* provides both static and dynamic environmental context. The static topology includes campus maps with roads, sidewalks, corridors, and intersections, while dynamic agents include pedestrians, other robots, and service vehicles. The LasVSim platform supports scenario generation, scenario replay, and large-scale parallel simulation, enabling interaction-rich environments to be reconstructed for evaluation. During each simulation step, environment feedback containing the ego robot state and surrounding agent information is delivered to downstream modules.

To ensure consistent interaction between the environment and learning-based components, a standardized state–action–reward interface is adopted. The *Interface and Data Abstraction Module* transforms raw environment feedback into structured representations suitable for prediction and decision-making. This layer performs ego robot state encoding, surrounding agent representation, prediction input formatting, and decision output adaptation, forming a unified data interface for downstream modules.

Based on the abstracted state representation, the *Trajectory Prediction Module* performs short-horizon multi-agent motion forecasting. The module generates multi-modal future trajectories of surrounding agents together with their associated mode probabilities. These predicted trajectories capture possible interaction outcomes in the near future and are explicitly passed to the decision-making module as forward-looking information.

The predicted trajectories are incorporated into a *Prediction-Conditioned Decision State*, which combines the current system state with the predicted future motions of surrounding agents. Within the *Self-Decision-Making Module*, candidate actions are evaluated through a prediction-aware cost evaluation mechanism that considers safety cost, task efficiency, motion smoothness, rule compliance, and action cost. A prediction-conditioned policy then selects the most appropriate behavior through risk-aware action evaluation and receding-horizon decision-making.

Finally, the selected action or motion primitive is executed by the *Motion Planning and Control Layer*, which converts high-level decisions into executable trajectories and low-level control commands. This layer performs trajectory tracking, collision avoidance, and motion control to ensure stable robot operation. The resulting robot motion and environment interaction are fed back into the simulation environment, forming a closed-loop system for evaluating prediction-aware decision-making under interaction-rich campus scenarios.

## 4. Trajectory Prediction-Enabled Self-Decision-Making

### 4.1. Trajectory Prediction Framework

**Figure 3:**
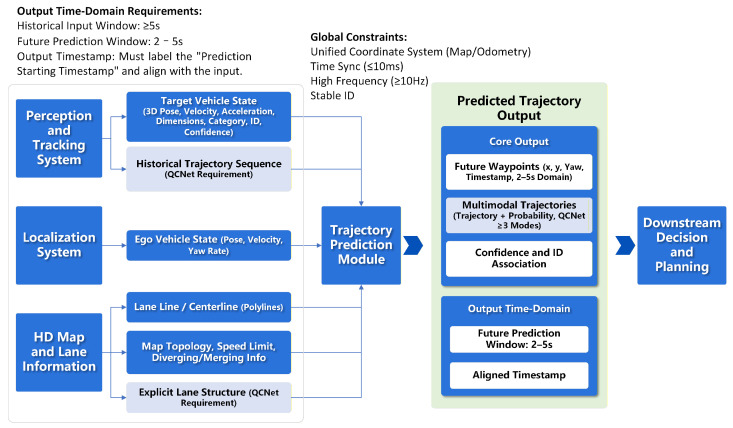
The trajectory prediction framework and Input–Output Flow.

The trajectory prediction component in this study follows a query-centric design, with QCNet adopted as the core prediction backbone [21]. As shown in Figure 3, the overall pipeline combines perception and tracking outputs, localization information, and high-definition map data into a unified prediction module operating under shared global constraints.

The perception and tracking system provides the current states of surrounding agents, including 3D position, velocity, acceleration, object dimensions, semantic category, confidence score, and persistent identifiers. For each target agent, we additionally construct a fixed-length historical trajectory sequence as required by QCNet. Historical trajectories are resampled onto a common time axis (≥10 Hz), and the input window is set to at least 5 s to retain sufficient temporal context for motion inference.

The localization module supplies the ego-vehicle state (pose, velocity, yaw rate), allowing for surrounding agents to be represented within a consistent coordinate frame. Road structure information is obtained from the high-definition map and lane modules, including lane centerlines, topology, speed limits, and merging or diverging semantics. Rather than relying solely on polyline representations, lane structures are encoded in a topological format compatible with QCNet’s structured input design. All signals are transformed into a unified coordinate frame (map or odometry) and temporally synchronized, with a maximum alignment tolerance of 10 ms to maintain spatial-temporal consistency across modules.

The prediction module outputs multi-modal future trajectories for each target agent. Each predicted trajectory consists of a sequence of future waypoints parameterized by position, yaw, and timestamp over a 2–5 s horizon, together with an associated probability. Output timestamps are aligned with a predefined prediction start time so that the results can be directly consumed by downstream decision-making and planning components. The overall training procedure of the QCNet-based model is summarized in Algorithm 1.
**Algorithm 1:** QCNet Training Procedure for Multi-Modal Trajectory Prediction
  **Input**: Historical trajectories **X**, lane topology graph G, ground-truth future
    trajectories $Y^{gt}$ (Let $Y^{gt}={\left\{y_{i}^{T_{h}+1:T_{h}+T_{f}}\right\}}_{i=1}^{N}$, denote the set of all
    ground-truth future trajectories);
  **Output**: Trained QCNet parameters θ;

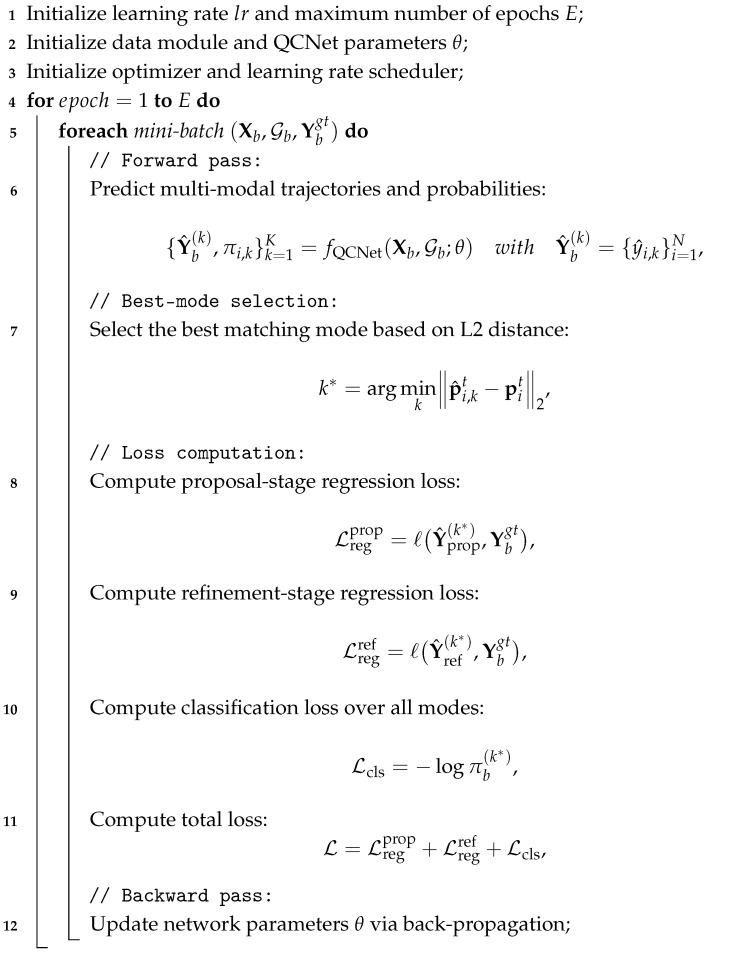



### 4.2. Trajectory Prediction Algorithm

To enable prediction-aware self-decision-making in campus environments, this work adopts a learning-based multi-agent trajectory prediction framework that forecasts the short-horizon future motions of surrounding dynamic agents, including pedestrians, service vehicles, and other robots. The prediction module provides probabilistic, multi-modal trajectory hypotheses that explicitly capture interaction uncertainty, serving as a forward-looking input for downstream decision-making.

#### 4.2.1. Problem Setting and Inputs

Let the environment contain an ego cleaning robot and a set of surrounding dynamic agents indexed by i∈A(|A|=N). For each agent *i*, a sequence of historical states over $T_{h}$ time steps is observed:

(5) $$x_{i}^{0:T_{h}}={\left(x_{i}^{t},y_{i}^{t},v_{i}^{t},\theta_{i}^{t}\right)}_{t=0}^{T_{h}}$$

where $\left(x_{i}^{t},y_{i}^{t}\right)$ denotes the planar position, $v_{i}^{t}$ the velocity, and $\theta_{i}^{t}$ the heading angle at time step *t*. All agent trajectories are represented in a unified global coordinate system aligned with the campus map.

The prediction task aims to estimate a distribution over each agent’s future trajectory over a horizon of $T_{f}$ steps:

(6) $$p\left(y_{i}^{T_{h}+1:T_{h}+T_{f}}|x_{1:N}^{0:T_{h}}\right)$$

where $y_{i}^{T_{h}+1:T_{h}+T_{f}}$ denotes the future positions (and optionally headings) of agent *i*, conditioned on the historical states of all agents.

#### 4.2.2. Query-Centric Multi-Agent Representation

To model heterogeneous and densely interacting agents in campus scenarios, a *query-centric representation* is adopted. Each target agent to be predicted is treated as a *query*, while all agents (including itself) provide contextual information. Historical motion cues are encoded through relative motion features, including displacement vectors, velocity magnitudes, and heading changes. For each agent, the motion feature vector is constructed as

(7) $$f_{i}^{t}=\left[∥\Delta p_{i}^{t}∥,\;\phi \left(\Delta p_{i}^{t},h_{i}^{t}\right),\;∥v_{i}^{t}∥,\;\phi \left(v_{i}^{t},h_{i}^{t}\right)\right]$$

where $\Delta p_{i}^{t}=p_{i}^{t}-p_{i}^{t-1}$ denotes the relative displacement, $v_{i}^{t}$ is the velocity vector, and $h_{i}^{t}=\left[\cos\theta_{i}^{t},\sin\theta_{i}^{t}\right]$ denotes the unit heading vector. The function $\phi \left(a,b\right)=\mathrm{atan}2\left(a_{x}b_{y}-a_{y}b_{x},a_{x}b_{x}+a_{y}b_{y}\right)$ computes the signed relative angle between two vectors. These continuous features are embedded via Fourier embeddings and combined with agent-type embeddings to form compact latent representations.

#### 4.2.3. Temporal and Interaction Encoding

The prediction model jointly captures *temporal evolution* and *inter-agent interactions* using a factorized attention mechanism.

**Temporal encoding** models dependencies across historical time steps of the same agent. For each agent, temporal edges are constructed between valid time steps within a predefined temporal span, and relative temporal features are encoded as

(8) $$r_{i}^{t,t^{\prime }}=\left[∥\Delta p_{i}^{t,t^{\prime }}∥,\;\phi \left(\Delta p_{i}^{t,t^{\prime }},h_{i}^{t}\right),\;\Delta \theta_{i}^{t,t^{\prime }},\;\Delta t\right]$$

where $\Delta p_{i}^{t,t^{\prime }}=p_{i}^{t}-p_{i}^{t^{\prime }}$ denotes the relative displacement between two time steps *t* and $t^{\prime }$ of the same agent, $\Delta \theta_{i}^{t,t^{\prime }}=\theta_{i}^{t}-\theta_{i}^{t^{\prime }}$ is the relative heading change, and $h_{i}^{t}=\left[\cos\theta_{i}^{t},\sin\theta_{i}^{t}\right]$ denotes the unit heading vector. The function ϕ(a, b) computes the signed relative angle between two vectors and is defined as Equation (7).

**Interaction encoding** captures spatial interactions between agents at the same time step. A distance-based graph is constructed, where edges connect agents within a predefined interaction radius $r_{\mathrm{int}}$. Relative interaction features are defined as

(9) $$r_{i,j}^{t}=\left[∥p_{i}^{t}-p_{j}^{t}∥,\;\phi \left(p_{i}^{t}-p_{j}^{t},h_{i}^{t}\right),\;\Delta \theta_{i,j}^{t}\right]$$

where $\Delta \theta_{i,j}^{t}=\theta_{i}^{t}-\theta_{j}^{t}$ denotes the relative heading difference between agent *i* and agent *j* at time step *t*.

Temporal attention and interaction attention are applied alternately, enabling each agent’s latent representation to aggregate both its motion history and nearby agents’ behaviors. This structure is particularly suited for campus environments, where frequent pedestrian crossings, slow-moving vehicles, and robot–human interactions coexist.

#### 4.2.4. Multi-Modal Trajectory Decoding

To reflect the inherent uncertainty of future motions in unstructured campus environments, the model predicts *multiple plausible future trajectories* for each agent. A set of *K* latent motion modes is initialized and refined through a two-stage decoding process.

In the proposal stage, each mode generates a coarse future trajectory using recurrent decoding over $T_{f}$ steps. Note that, $\left(x_{i}^{t},y_{i}^{t}\right)$ is equivalently denoted as $p_{i}^{t}$ in the following sections, and thus

(10) $${\hat{y}}_{i,k}^{\left(p\right)}={\left\{{\hat{p}}_{i,k}^{t}\right\}}_{t=T_{h}+1}^{T_{h}+T_{f}},\;k=1,\ldots ,K$$

In the refinement stage, the proposed trajectories are further adjusted using cross-attention between modes, temporal context, and interacting agents, yielding refined predictions:

(11) $${\hat{y}}_{i,k}^{\left(r\right)}={\hat{y}}_{i,k}^{\left(p\right)}+\Delta {\hat{y}}_{i,k}$$

Each mode is associated with a probability $\pi_{i,k}$, forming a mixture distribution over future trajectories:

(12) $$p\left(y_{i}\right)=\sum_{k=1}^{K}\pi_{i,k}p_{k}\left(y_{i}\right)$$

This multi-modal output is critical for downstream decision-making, as it enables the cleaning robot to reason about alternative behaviors of pedestrians or other robots (e.g., yielding, crossing, or continuing straight).

#### 4.2.5. Training Objective

During training, the model jointly optimizes trajectory accuracy and mode probability estimation in a multi-modal prediction setting. For each agent, the prediction head outputs *K* candidate future trajectories together with their corresponding mode probabilities. To supervise the multi-modal outputs, a best-matching mode selection strategy is adopted. Specifically, for each agent *i*, the mode index $k^{*}$ that best matches the ground-truth trajectory is determined by minimizing the cumulative $\ell_{2}$ distance over the prediction horizon:

(13) $$k^{*}=\arg\min_{k}\sum_{t}{∥{\hat{p}}_{i,k}^{t}-p_{i}^{t}∥}_{2}$$

where ${\hat{p}}_{i,k}^{t}$ denotes the predicted position of the *k*-th trajectory mode at time step *t*, and $p_{i}^{t}$ is the corresponding ground-truth position.

The overall training loss consists of two complementary terms: a regression loss that enforces trajectory accuracy for the selected mode, and a classification loss that encourages the model to assign higher probability to the best-matching mode:

(14) $$L=L_{\mathrm{reg}}\left({\hat{y}}_{i,k^{*}},y_{i}\right)+L_{\mathrm{cls}}\left(\pi_{i},k^{*}\right)$$

The regression loss $L_{\mathrm{reg}}$ consists of two stages, $L_{\mathrm{reg}}=L_{reg}^{prop}+L_{reg}^{ref}$, and it is applied only to the selected mode $k^{*}$, following a winner-takes-all strategy, while the classification loss $L_{\mathrm{cls}}$ is implemented as a cross-entropy loss over the predicted mode probabilities $\pi_{i}$. This formulation encourages each mode to specialize in different plausible futures, thereby promoting trajectory diversity while maintaining prediction accuracy.

As a result, the trained prediction model produces a set of probabilistic future trajectories for all relevant agents. These predictions are not treated as hard constraints, but rather as anticipatory inputs that inform the downstream self-decision-making module about potential conflicts, safe gaps, and interaction risks. The integration of prediction and decision-making is detailed in the subsequent section.

### 4.3. Integration with Self-Decision-Making

This section describes how the predicted trajectories of surrounding agents are explicitly incorporated into the self-decision-making framework, enabling anticipatory and interaction-aware decisions for autonomous cleaning robots in campus environments. Note that ℓ(·) denotes the instantaneous decision cost, while L(·) represents the training loss used in the prediction module.

#### 4.3.1. Prediction-Conditioned Decision State

At each decision step *t*, the self-decision-making module operates on an augmented state that includes both current observations and predicted future motions:

(15) $${\tilde{s}}_{t}=\left(s_{t},{\hat{T}}_{t}\right)$$

where $s_{t}$ denotes the current system state (ego robot state, surrounding agents, and local map context), and

(16) $${\hat{T}}_{t}=\left\{{\hat{\tau }}_{t}^{\left(i\right)}|i=1,\ldots ,N\right\}$$

represents the set of predicted future trajectories of surrounding agents, where ${\hat{\tau }}_{t}^{\left(i\right)}$ corresponds to the most likely trajectory mode selected according to the predicted mode probabilities.

This formulation allows for the decision-making module to reason over both instantaneous and future interaction states in a unified manner. Further, although the prediction module produces multi-modal future trajectories, only the most probable mode is used to construct the decision state for computational efficiency.

#### 4.3.2. Prediction-Aware Action Evaluation and Safety Constraint

Let $a_{t}\in A$ denote a candidate action or short-horizon motion primitive of the ego robot. The quality of a candidate action is evaluated through a cost function conditioned on predicted trajectories:

(17) $$J\left(a_{t}|{\tilde{s}}_{t}\right)=E\left[\sum_{k=0}^{H_{d}}\ell \left(s_{t+k},a_{t+k},{\hat{T}}_{t+k}\right)\right]$$

The instantaneous cost ℓ(·) is decomposed into multiple components:

(18) $$\ell =\ell_{\mathrm{safe}}+\ell_{\mathrm{task}}+\ell_{\mathrm{smooth}}+\ell_{\mathrm{rule}}$$

where each term captures a specific decision objective.

Safety is enforced by evaluating the spatial relationship between the ego robot’s trajectory and the predicted future positions of surrounding agents. For agent *i* and future step *k*, the predicted relative distance is defined as

(19) $$d_{i,k}=\left|\right|p_{\mathrm{ego}}^{t+k}-{\hat{p}}_{i}^{t+k}{\left|\right|}_{2}$$

In addition, a deterministic safety cost for single-trajectory case (or constraint) is then imposed as

(20) $$\ell_{\mathrm{safe}}^{\det}=\sum_{i=1}^{N}\sum_{k=1}^{K}\phi \left(d_{i,k}\right)$$

where ϕ(·) is a distance-based safety penalty function that increases rapidly when the distance between agents becomes smaller than a safety threshold. In this work, the penalty function is defined as

(21) $$\phi \left(d\right)=\left\{\begin{array}{cc} -\log\left(\frac{d}{d_{s}}\right), & d<d_{s}\\ 0, & d\geq d_{s} \end{array}\right.$$

where $d_{s}$ denotes the safety distance threshold. This logarithmic barrier function ensures that no penalty is introduced when the predicted distance is above the safety margin, while potential collision risks are penalized increasingly as the distance decreases.

#### 4.3.3. Risk-Aware and Multi-Modal Integration

When multi-modal predictions are available, the expected safety and interaction cost is computed by marginalizing over motion modes:

(22) $$\ell_{\mathrm{safe}}^{\exp}=\sum_{i=1}^{N}\sum_{m=1}^{M}\sum_{k=1}^{K}\pi_{i,m}\;\phi \;\left(∥p_{\mathrm{ego}}^{t+k}-{\hat{p}}_{i,m}^{t+k}{∥}_{2}\right)$$

where the ${\hat{p}}_{i,m}^{t+k}$ denotes the predicted position under mode *m* with probability $\pi_{i,m}$. The same penalty function ϕ(·) defined above is used to evaluate safety costs under each predicted motion mode. Specifically, $\ell_{\mathrm{safe}}^{\exp}$ reduces to $\ell_{\mathrm{safe}}^{\det}$ when only a single prediction mode is considered. This expectation-based formulation enables risk-sensitive decision-making under prediction uncertainty. Note that while only the most probable mode is used to construct the augmented decision state, all predicted modes are considered when evaluating risk-aware safety cost.

#### 4.3.4. Prediction-Enabled Policy Formulation

From a policy perspective, the decision-making process can be expressed as a prediction-conditioned policy:

(23) $$\pi (a_{t}|{\tilde{s}}_{t})$$

where ${\tilde{s}}_{t}=\left(s_{t},{\hat{T}}_{t}\right)$ denotes the prediction-augmented decision state. Thus, Equation (23) maps the augmented state to an action distribution. During training, the policy is optimized to minimize the expected cumulative cost:

(24) $$\min_{\pi }\;E_{\pi }\left[\sum_{t=0}^{T}J\left(a_{t}|{\tilde{s}}_{t}\right)\right]$$

where $J(a_{t}|{\tilde{s}}_{t})$ is the prediction-conditioned action cost defined in Equation (17). This formulation applies to both optimization-based planners and learning-based decision policies. In practice, the policy is trained offline using interaction-rich scenarios and executed online in a receding-horizon manner to ensure real-time performance.

Importantly, predicted trajectories are not treated as hard constraints, but as probabilistic and time-indexed cues that influence decision evaluation. By embedding prediction results into both the cost function and the policy conditioning, the proposed framework enables proactive yet conservative decisions, balancing safety, task continuity, and motion stability in dynamic campus environments. Although the trajectory prediction module generates multiple possible future trajectories, only the most probable mode is incorporated into the decision state to maintain computational efficiency and ensure real-time planning performance. While this simplification reduces the dimensionality of the decision state, it may limit the ability of the system to fully capture prediction uncertainty in highly interactive scenarios. In principle, full multi-modal predictions could be incorporated into the decision-making process through probabilistic risk evaluation or scenario-based planning strategies, allowing for the robot to consider multiple possible interaction outcomes. Exploring such uncertainty-aware decision mechanisms represents an important direction for future research.

## 5. Experimental Evaluation and Conclusions

### 5.1. Experimental Setup

The proposed trajectory prediction-enabled self-decision-making framework is evaluated through extensive simulation-based experiments. The experiments are conducted using real-world driving data and a high-fidelity simulation environment to assess both prediction accuracy and decision-making performance under interaction-rich scenarios. The parameters of the simulated cleaning robots can be seen in Table 1.

For trajectory prediction evaluation, the model is trained and tested on both public benchmarks and real-world vehicle data. The public dataset follows the *Argoverse2* protocol, where each scenario contains 5 s of historical observations and up to 6 s of future trajectories. The dataset is split into training, validation, and test sets in a ratio of 8:1:1. For real-world evaluation, large-scale vehicle trajectory data collected from complex scenarios are processed using a unified data generation pipeline, including coordinate projection, temporal alignment, trajectory filtering, and smoothing.

The prediction model is implemented using PyTorch 2.0.1 and PyTorch Lightning 2.0.9, and trained on NVIDIA A100 GPUs (Nvidia, Santa Clara, CA, USA) (see Table 2 for detailed training configuration). Different prediction horizons 3 s, 5 s, and 6 s and multiple modality settings are evaluated to analyze the trade-off between prediction accuracy and temporal coverage. The prediction model is built upon the QCNet architecture, which adopts a query-centric design to decouple scene encoding from trajectory decoding and enables efficient multi-modal long-horizon prediction. An ablation study is conducted on the number of encoder layers under identical training settings. The results show that using two encoder layers achieves the best overall performance in terms of minADE, minFDE, and MR (see the next section for the explanation), providing a favorable balance between accuracy and model complexity. Therefore, a two-layer encoder configuration is adopted in all experiments. Further architectural details of QCNet are referred to the original paper.

To evaluate the impact of prediction on downstream decision-making, a simulation-based planning and decision framework is constructed. The environment includes multi-lane road segments, interaction with surrounding vehicles, and stochastic traffic flow generated using rule-based driver models. Three planning strategies are compared: (1) lane-following with longitudinal control, (2) rule-based lane-change decision with longitudinal planning, and (3) the proposed prediction-aware self-decision-making framework. Each configuration is tested over multiple randomized runs, and performance metrics are averaged across trials.

### 5.2. Evaluation Metrics

The experimental evaluation adopts both trajectory prediction metrics and decision-making performance metrics.

To comprehensively evaluate the trajectory prediction performance, we adopt several widely used metrics in the trajectory prediction literature as follows:

- **Minimum Average Displacement Error (minADE)** [28,29]: The average Euclidean distance between the predicted trajectory and the ground truth trajectory over the prediction horizon, computed for the best prediction mode;
- **Minimum Final Displacement Error (minFDE)** [28,29]: The Euclidean distance between the predicted final position and the ground truth final position;
- **Minimum Average Heading Error (minAHE)** [30]: The average angular deviation between the predicted heading angles and the ground truth heading angles over the prediction horizon, computed for the best prediction mode;
- **Minimum Final Heading Error (minFHE)** [30]: The angular deviation between the predicted final heading angle and the ground truth heading angle at the final prediction timestep.
- **ADE Rate (ADERate)** [31]: The proportion of prediction instances where
  (25) $$\mathrm{minADE}<\epsilon_{\mathrm{ADE}}$$
- **FDE Rate (FDERate)** [31]: The proportion of prediction instances where
  (26) $$\mathrm{minFDE}<\epsilon_{\mathrm{FDE}}$$

In addition to the above-mentioned common trajectory prediction metrics such as minADE and minFDE, which measure the spatial deviation between predicted trajectories and ground-truth trajectories, we additionally evaluate heading prediction accuracy using minAHE and minFHE. These heading-based metrics quantify the angular deviation between predicted and ground-truth orientations, which is particularly important for mobile robots operating in dynamic environments where both position and orientation influence motion feasibility and decision-making. Furthermore, to assess the reliability of the predicted trajectories, we also report ADERate and FDERate, which measure the proportion of predictions whose displacement errors fall within predefined thresholds. These metrics provide an additional perspective on prediction robustness and practical usability in navigation and planning tasks.

In addition, behavior-level metrics are introduced to evaluate the correctness of maneuver prediction, such as lane-change detection accuracy, which is determined by analyzing the predicted trajectory relative to lane boundaries.

For decision-making and planning evaluation, the following metrics are employed:

- **Average Speed**:
  (27) $$V=\frac{D}{T}$$
  where *D* is the total travel distance and *T* is the effective travel time.
- **Energy Consumption** [32]:
  (28) $$E=\sum_{i}3.25\cdot e^{0.01v_{i}}\cdot \frac{d_{i}}{100}$$
  where $v_{i}$ and $d_{i}$ denote the speed and distance of the *i*-th segment.
- **Minimum Distance to Leading Vehicle**: The minimum longitudinal distance maintained between the ego vehicle and the preceding vehicle during each run.
- **Comfort**: Measured by the mean rate of change of lateral acceleration:
  (29) $$C=\frac{1}{T}\sum_{t}\left|{\dot{a}}_{y}\left(t\right)\right|$$

These metrics jointly reflect efficiency, safety, energy consumption, and ride comfort.

### 5.3. Results and Discussion

#### 5.3.1. Offline Training

As mentioned above, we first conducted experiments on the public Argoverse2 dataset. The dataset contains 249,880 traffic scenarios. Next, we evaluated the influence of encoder depth on trajectory prediction performance. After training for 10 epochs, the model with two encoder layers achieved the best results, with a minADE of 0.73, a minFDE of 1.27, and a Miss Rate (MR) of 0.16, as shown in Table 3.

In addition, we investigated the impact of different prediction horizons on trajectory prediction accuracy. The prediction horizons were set to 3 s, 4 s, and 5 s. As shown in Table 4, increasing the prediction horizon leads to larger displacement errors (minADE and minFDE), while the decision-oriented success metrics (ADERate and FDERate) remain relatively stable. These results indicate that longer prediction horizons introduce greater uncertainty without significantly improving decision-related outcomes in the Argoverse2 dataset.

To assess the generalization capability of the proposed model, we fine-tune the network trained on public datasets using a real-world vehicle trajectory dataset. All trajectories are represented in the Gauss–Krüger projected coordinate system to maintain metric consistency for spatial modeling. The training set contains 79,283 samples, while the validation and test sets include 11,892 and 7931 samples, respectively. In total, the dataset corresponds to approximately 302 h of recorded driving data. All evaluation metrics reported in this study are averaged over the entire validation or test dataset, which contains a large number of trajectory samples across diverse traffic scenarios. As a result, the aggregated metrics provide statistically stable and representative estimates of model performance, as the averaging process effectively reduces the impact of individual sample variability. Therefore, the observed performance differences reflect consistent trends across the dataset rather than random fluctuations, indicating reliable and robust evaluation outcomes.

**Figure 4:**
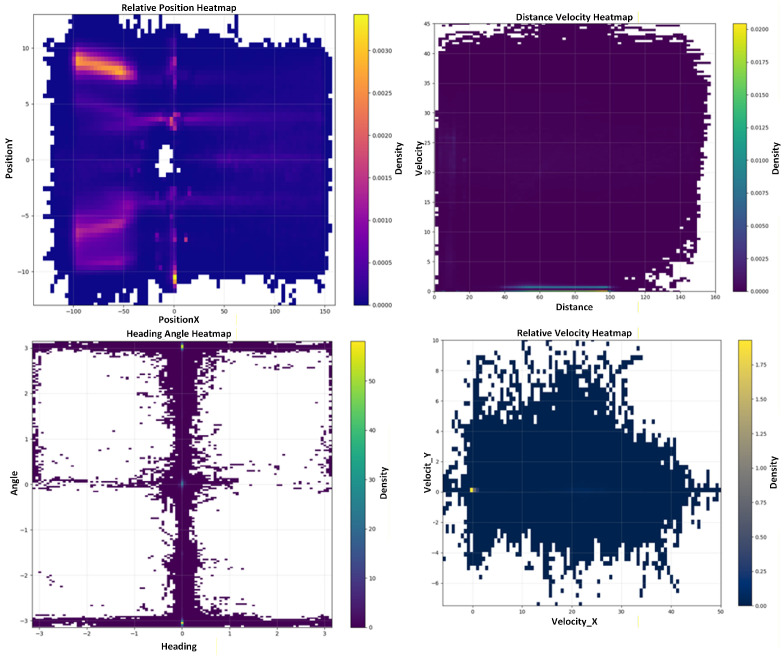
The distribution of a real-world vehicle trajectory dataset.

Figure 4 presents the overall data distribution in the Gauss–Krüger coordinate frame, including spatial position, velocity, heading angle, and distance-related statistics. The dataset covers a wide range of spatial and kinematic conditions observed in real driving, providing sufficient diversity for both training and evaluation.

Quantitative evaluation results are summarized in Table 5. On the validation set, the model achieves an ADE of 92.31%, while on the test set the ADE is 91.90%. The minimum final displacement error (minFDE) is 1.39 m on the validation split and 1.46 m on the test split. Lane-changing behavior prediction accuracy is approximately 79% on both datasets.

Table 6 summarizes the quantitative results of trajectory prediction and behavior prediction under different temporal and spatial horizons. Overall, the proposed model achieves consistently strong performance on both the validation and test sets, indicating good generalization capability across different prediction settings. A clear trend can be observed that, as the prediction horizon becomes shorter, both the ADERate and FDERate increase, reflecting improved accuracy in short-term trajectory forecasting. This phenomenon is consistent with the inherent uncertainty accumulation in long-term motion prediction: as the prediction horizon extends, the number of possible interaction outcomes among agents increases, making accurate trajectory estimation more challenging.

In contrast, the accuracy of lane-changing behavior prediction remains relatively stable across different prediction distances, suggesting that the proposed model is less sensitive to spatial horizon variations in behavior-level prediction. These results demonstrate that the model can effectively capture both fine-grained trajectory evolution and high-level driving behaviors under varying prediction horizons. More importantly, since most interaction-critical events in campus environments occur within a short temporal window (e.g., yielding, local avoidance, or speed adjustment), reliable short-horizon prediction provides sufficiently accurate cues for downstream decision-making. Therefore, the experimental results further support the design choice of using short-horizon trajectory prediction to guide the decision-making module in the proposed framework.

Table 7 presents the lane-changing behavior prediction accuracy for predicting whether a lane change occurs within a distance of *s* meters ahead of the ego vehicle under a fixed trajectory prediction horizon of 6 s. As the prediction distance increases, the behavior prediction accuracy first decreases and then slightly increases. For distances below 100 m, the reduction in accuracy is mainly caused by accumulated trajectory prediction errors over longer spatial horizons, which negatively affect behavior inference. Beyond 100 m, lane geometry modeled by cubic polynomial fitting becomes the dominant factor, making lane-changing decisions less sensitive to trajectory prediction errors and leading to a modest recovery in prediction accuracy.

Overall, the experimental results demonstrate that the proposed approach maintains stable and competitive prediction performance when transferred from public datasets to real-world vehicle data. The model is able to reliably forecast future agent trajectories across diverse and complex driving scenarios, confirming its robustness and practical applicability for offline trajectory prediction in real-vehicle settings.

#### 5.3.2. Online Planning

The online planning performance was evaluated using the LasVSim simulation platform, a large-scale autonomous driving simulation environment designed for high-fidelity scenario generation and interaction testing. The platform provides a full-stack simulation framework that integrates map construction, intelligent traffic flow generation, vehicle dynamics modeling, perception–decision–control pipelines, V2X communication, and performance evaluation modules. LasVSim supports heterogeneous traffic participants, including cleaning robots, passenger vehicles, bicycles, and pedestrians, which are controlled by an AI-driven traffic behavior model. The traffic model is trained using real-world trajectory data combined with generative data augmentation, enabling diverse and interactive behaviors such as lane merging, intersection negotiation, and mixed human–vehicle interactions. Compared with traditional rule-based traffic flow models, this approach produces more realistic and adaptive interaction patterns among agents.

**Figure 5:**
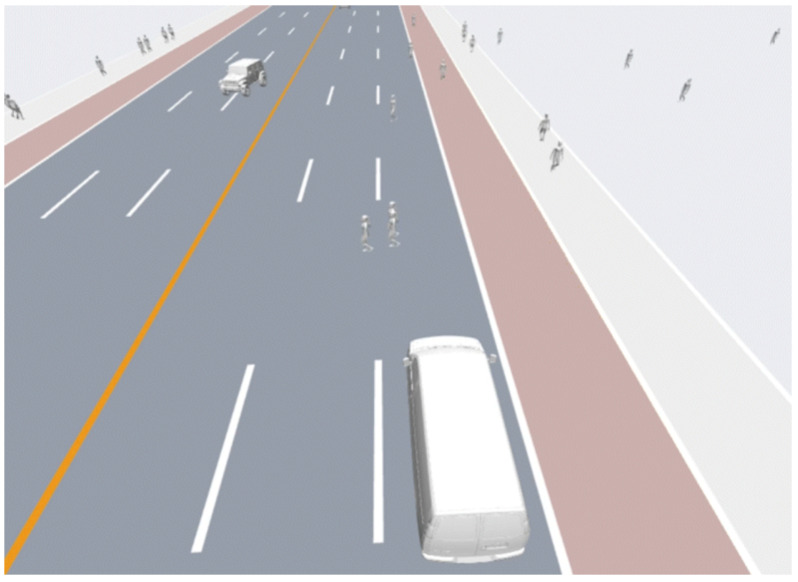
The LasVSim simulation platform.

Scenario environments are constructed using semantic road maps that describe corridors, intersections, and campus road networks. During simulation initialization, agents are assigned randomized positions, velocities, and navigation goals based on map semantics, creating diverse interaction situations for evaluation. The platform further supports large-scale parallel simulation, enabling thousands of agents to run simultaneously and allowing for efficient testing of interaction-rich scenarios that would be difficult to reproduce in real-world experiments (Figure 5).

In online planning, the proposed framework was designed to operate under the same update frequency as the practical sensing and planning pipeline, namely 10 Hz, corresponding to a control cycle of approximately 100 ms. In the current study, the system is evaluated using the LasVSim platform, which provides a replay-based simulation environment for algorithm validation rather than a real-time embedded deployment platform. Consequently, we do not report a strictly measured end-to-end runtime latency for the integrated prediction and decision-making pipeline on target hardware. However, as an indirect indication of computational efficiency, the prediction module (cf. Section 5.3.1) achieves approximately 10.66 validation batches per second with a batch size of 16 in the offline evaluation setting, corresponding to about 170 scenes per second in batched inference. This result suggests that the prediction network itself is computationally lightweight relative to the 10 Hz operating requirement.

**Figure 6:**
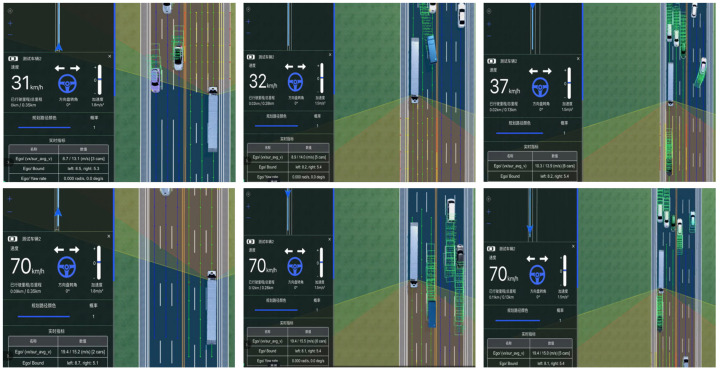
Planning in campus scenarios with varying densities.

We further assess performance under traffic flows with different densities (Figure 6). In the absence of surrounding vehicles, the ego vehicle accelerates from 30 km/h to a target speed of 70 km/h, while the steering angle remains close to 0° during straight-line motion. Although actual campus operating speeds are typically limited to 30 km/h, higher target speeds are introduced in simulation to evaluate the robustness of the control and planning framework under more demanding dynamic conditions. The vehicle speed converges smoothly toward the reference value without noticeable oscillation. Under low-density traffic conditions (0–300 pcu/h/ln), surrounding vehicles are sparse and maintain relatively large headways. The ego vehicle follows the predefined acceleration profile and reaches the cruising speed with minimal disturbance. Since braking or lane-change maneuvers are rarely required, longitudinal control remains stable and tracking error remains small. In medium-density traffic scenarios (0–700 pcu/h/ln), interactions occur more frequently. Speed adjustments are occasionally required due to leading vehicles or adjacent lane changes. During acceleration, minor speed fluctuations can be observed; however, the vehicle gradually stabilizes near the desired cruising speed once traffic conditions allow.

**Figure 7:**
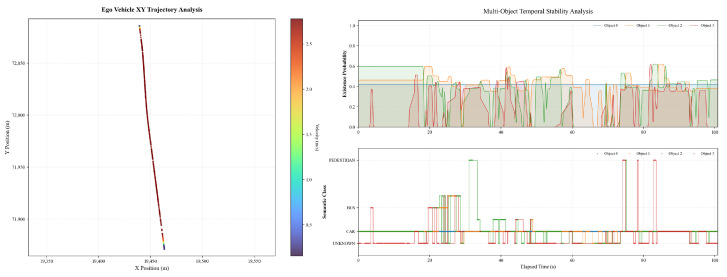
The ego vehicle trajectory and perception results in a straight-driving scenario.

We then further verified the proposed self-decision-making framework in a real campus scenario. Figure 7 shows the ego vehicle trajectory and perception results during a representative straight-driving scenario. The XY trajectory remains smooth and nearly linear, with the ego vehicle traveling at an approximately constant cruising speed of about 2.6–2.8m/s over most of the path, indicating the absence of significant lateral maneuvers or replanning events. The color-coded trajectory further confirms limited speed variation during steady motion. The temporal evolution of multi-object perception outputs demonstrates continuous detection of several obstacles, whose existence probabilities generally remain within the range of approximately 0.3 to 0.6. Although occasional short-term confidence fluctuations and transient object classification changes are observed, particularly for lower-confidence targets, the overall perception results remain stable and do not induce noticeable disturbances in the planning process under nominal straight-driving conditions.

**Figure 8:**
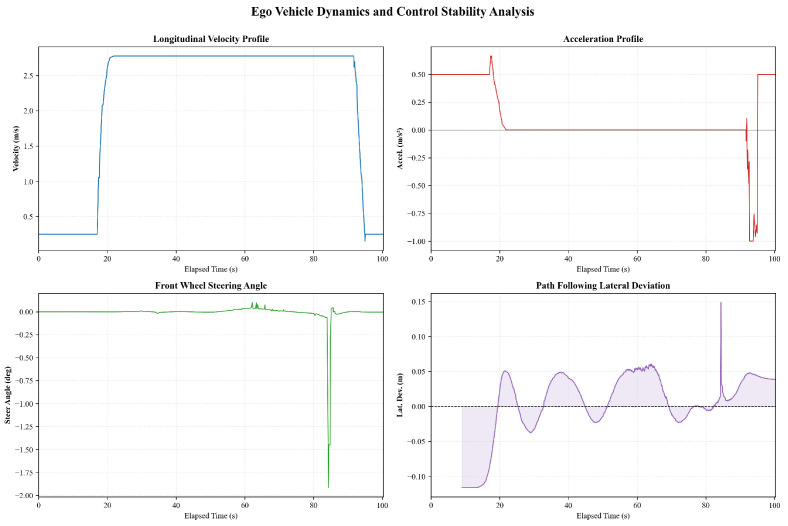
The ego vehicle dynamics and control performance in a straight-driving scenario.

The corresponding ego vehicle dynamics and control performance are illustrated in Figure 8. After a short acceleration phase lasting approximately 2–3s, the longitudinal velocity converges to a steady cruising value, while the acceleration signal remains close to zero for most of the driving interval. The peak longitudinal acceleration during the initial speed-up phase is bounded within approximately 0.5–$0.6\;m/s^{2}$, and the deceleration magnitude near the end of the segment remains below about $1.0\;m/s^{2}$. The steering angle stays near zero for the majority of the trajectory, consistent with straight-line motion, with only brief transient corrections reaching approximately 1.5–$2.0^{{}^{\circ}}$. Correspondingly, the lateral deviation from the reference path is confined within a small range of roughly ±0.1m and rapidly returns to nominal values after short-lived peaks. Overall, both longitudinal and lateral control responses exhibit stable and well-damped behavior, providing a reliable baseline for subsequent analysis of interaction-rich and prediction-aware driving scenarios.

**Figure 9:**
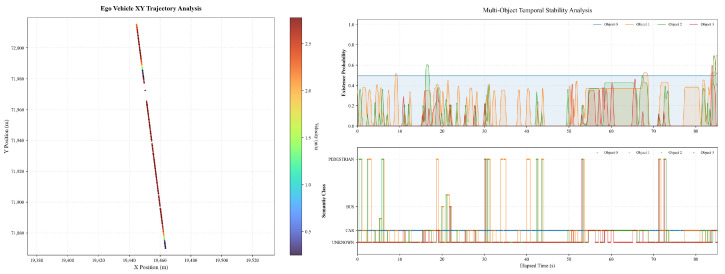
The ego vehicle trajectory and perception results with lane change inhibition.

Figure 9 illustrates the ego vehicle trajectory and perception results in a straight-driving scenario where a lane-change maneuver is initially prepared but eventually suppressed due to detected obstacles. The XY trajectory remains smooth and largely linear, with no evident lateral displacement indicative of an executed lane change. The ego vehicle maintains a stable cruising motion along the reference lane, with the trajectory points exhibiting minimal lateral spread throughout the observed interval. The perception results indicate the presence of multiple surrounding objects with time-varying existence probabilities. As shown in the temporal stability analysis, several objects exhibit intermittent detection with existence probabilities fluctuating predominantly within the range of approximately 0.1 to 0.6. Meanwhile, object classification labels remain mostly consistent, with the majority of detected objects categorized as vehicles. These perception signals coincide temporally with the suppression of the lane-change behavior, suggesting that the detected obstacles provide sufficient constraints to inhibit the lane-change decision under the current conditions.

**Figure 10:**
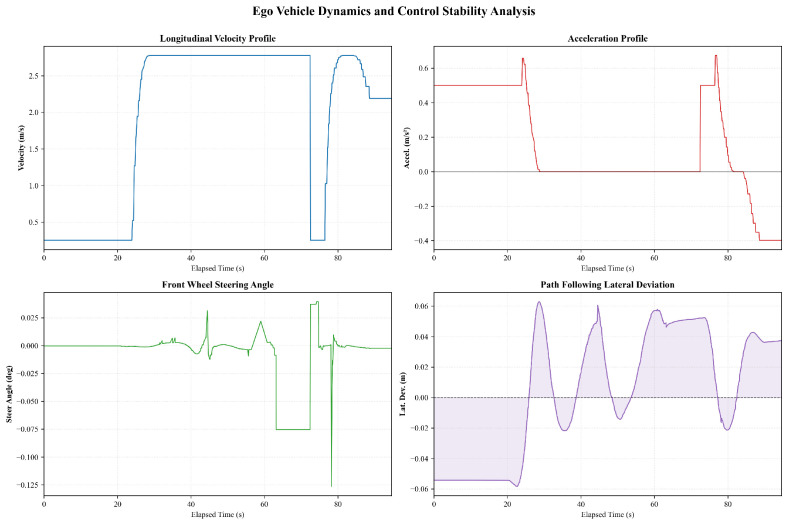
The ego vehicle dynamics and control performance with lane change inhibition.

The corresponding ego vehicle dynamics and control responses are presented in Figure 10. After an initial acceleration phase, the longitudinal velocity converges to a cruising speed of approximately 2.7m/s and remains stable for an extended period. A brief deceleration is observed around t≈70s, during which the velocity drops to below 0.5m/s before recovering, accompanied by bounded acceleration peaks within approximately $\pm 0.6\;m/s^{2}$. The steering angle remains close to zero for most of the trajectory, with short-duration deviations not exceeding about $0.1^{{}^{\circ}}$, indicating that no sustained lateral maneuver is executed. The lateral deviation from the reference path is confined within approximately ±0.06m, with transient oscillations that rapidly decay. Overall, the ego vehicle exhibits stable longitudinal and lateral control behavior, while the lane-change intention is effectively suppressed in response to obstacle-related perception cues.

**Figure 11:**
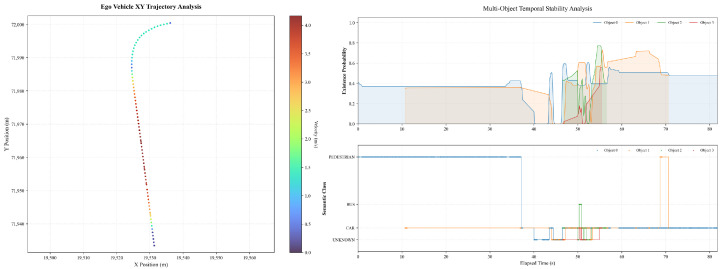
The ego vehicle trajectory and perception results at an unsignalized intersection.

Figure 11 presents the ego vehicle trajectory and perception outputs during a right-turn maneuver at an unsignalized campus intersection. The XY trajectory forms a curved path with continuous heading variation, consistent with a completed right turn rather than straight-line motion. Compared with straight-driving segments, the surrounding environment during the turn exhibits higher interaction density and less structured traffic organization, which is reflected in the perception results. Multiple objects are detected near the intersection area, and their existence probabilities vary over time, particularly during the turning phase (t≈45–60s). Intermittent transitions among vehicle-related categories are observed, which may be attributed to occlusions, limited observation duration, and dynamically changing viewpoints commonly encountered in unsignalized campus intersections.

**Figure 12:**
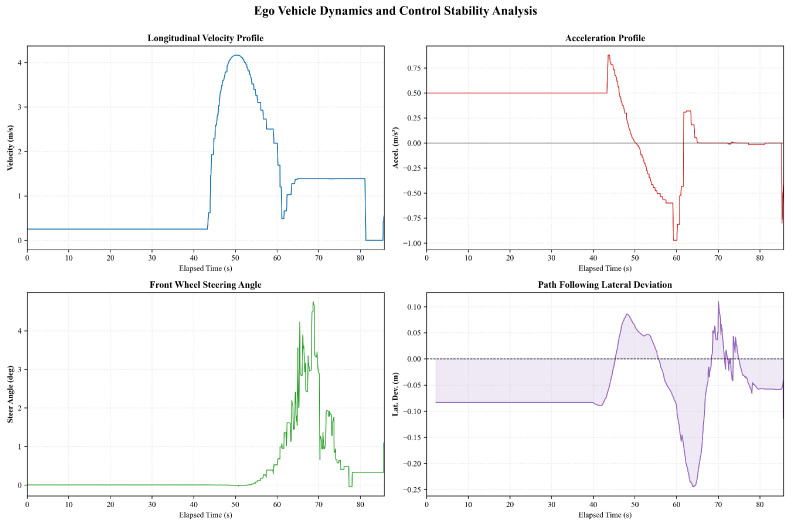
The ego vehicle dynamics and control performance at an unsignalized intersection.

The corresponding ego vehicle dynamics and control responses are illustrated in Figure 12. Prior to entering the intersection, the longitudinal velocity decreases from cruising level to below 1.0m/s, accompanied by sustained negative acceleration reaching approximately $-1.0\;m/s^{2}$. During the turning phase, the steering angle increases and peaks at around $4^{{}^{\circ}}$, while the lateral deviation reaches approximately −0.25m before gradually converging toward the reference path after the turn. Although longitudinal deceleration and lateral steering are strongly coupled during this maneuver, the control responses remain within bounded ranges throughout the interaction.

The real-vehicle experiments show that short-horizon multi-modal trajectory prediction can be incorporated into the decision-making loop, allowing for the vehicle to anticipate interactions and adjust its motion accordingly. In comparison with prediction-agnostic strategies, the proposed method produces smoother velocity profiles and reduces abrupt control variations during interactions with pedestrians and other moving agents. Throughout the experiments, no safety-critical events or manual interventions were recorded, and key indicators such as relative distance evolution and speed variation remained within acceptable ranges.

These observations suggest that effective decision-making does not necessarily require highly accurate long-horizon forecasting. Instead, reliable short-term prediction, when properly integrated into the control framework, appears sufficient to support stable and consistent behavior in real-world autonomous operation.

## 6. Conclusions

This work presents a self-decision-making framework for autonomous cleaning robots that incorporates multi-agent trajectory prediction within interaction-rich campus environments. By embedding predicted trajectories into the decision loop, the robot can account for short-term future interactions instead of relying solely on instantaneous perception. The framework connects perception-level forecasting with behavior selection in a modular manner that supports prediction-aware operation.

Offline and online evaluations were performed to assess the proposed approach. The offline experiments covered different temporal and spatial settings, while the online simulations examined vehicle behavior under varying traffic densities. Across these tests, the robot maintained stable speed tracking and handled interactions without safety-critical events. The experimental observations suggest that accurate long-horizon forecasting is not strictly necessary for effective decision-making in campus scenarios. Short-horizon prediction, when properly integrated into the control framework, appears sufficient to support stable and consistent autonomous operation in interaction-dense environments. Although the proposed framework demonstrates promising results in the simulation-based campus scenarios, several limitations remain. First, the current evaluation relies on a high-fidelity simulation platform where perception inputs are relatively clean compared with real-world sensing systems. In practical deployments, perception noise, missed detections, and occlusions caused by surrounding agents or environmental structures may affect the quality of trajectory prediction and decision-making. Second, the behavioral models of surrounding agents in simulation are still simplified compared with real human behaviors and service vehicle operations. Third, the runtime constraints of onboard computing systems and control execution latency of physical robotic platforms are not fully captured in the current simulation environment. These factors may influence the real-world performance of the proposed framework and highlight the need to further investigate the sim-to-real gap in future deployments.

Future work will focus on several technical directions to further extend the proposed framework. First, richer semantic representations will be incorporated into the interaction modeling process by integrating map semantics, behavioral context, and task-related information, enabling the prediction module to better capture environment structure and agent intent. Second, uncertainty-aware decision-making mechanisms will be introduced to explicitly account for prediction uncertainty, for example by incorporating probabilistic trajectory distributions or risk-sensitive cost evaluation into the decision layer. Third, the framework will be deployed on physical autonomous cleaning robot platforms to conduct long-term experiments in real campus environments, allowing for the system to be validated under realistic perception noise, occlusions, hardware latency, and runtime constraints of onboard computing systems. In particular, a full end-to-end runtime benchmark of the integrated prediction and decision-making pipeline on the target real-time deployment platform will be conducted as part of this future work. Finally, due to its modular architecture, the proposed framework can be extended to other mobile robotic systems that require prediction-aware decision-making in densely interactive environments, such as service robots and autonomous delivery platforms.

## Figures and Tables

**Table 1:** Physical and kinematic parameters of the autonomous cleaning robots.

Parameter	Symbol/Description	Value
Wheel radius	$r_{w}$ (radius of wheel)	0.522 m
Wheel width	$w_{w}$ (tire width)	0.295 m
Wheelbase	$L_{\mathrm{wb}}$ (front to rear wheel center distance)	5.3 m
Wheel tread	$T_{w}$ (left to right wheel center distance)	1.92 m
Front overhang	$O_{f}$ (Distance from front axle to vehicle front)	1.4 m
Rear overhang	$O_{r}$ (Distance from rear axle to vehicle rear)	2.02 m
Left overhang	Distance from left wheel to vehicle boundary	0.175 m
Right overhang	Distance from right wheel to vehicle boundary	0.175 m
Vehicle height	Overall vehicle height	2.972 m
Maximum steering angle	$\delta_{\max}$ (front wheel)	0.6 rad

**Table 2:** Training and development environment configuration.

Category	Parameter/Description	Value
Hardware	GPU model	NVIDIA A100-PCIE-40GB
	GPU memory	40 GB
	CUDA cores	6912
	Tensor cores	432
	Power consumption	250 W
	Memory bandwidth	1555 GB/s
	Architecture	Ampere
Software	Python environment	Python 3.8.19
	PyTorch version	2.0.1
	CUDA version	11.8
	Operating system	Linux 4.19.90
	Platform architecture	ARM64

**Table 3:** Impact of encoder depth on trajectory prediction performance.

Encoder Depth	minADE	minFDE	MR
0	0.76	1.33	0.18
1	0.74	1.30	0.17
2	0.73	1.27	0.16

**Table 4:** Impact of prediction horizon on trajectory prediction metrics.

Prediction Horizon	minADE	minFDE	ADERate	FDERate
3 s	0.26	0.44	0.99	0.98
4 s	0.38	0.67	0.99	0.95
5 s	0.54	0.95	0.98	0.90

**Table 5:** Performance evaluation on the real-world vehicle dataset.

Dataset Split	Metric	Value
Validation set	ADE Rate	0.9231
	minAHE	0.2848
	minFDE	1.3930
	Lane-changing accuracy	0.7927
Test set	ADE Rate	0.9190
	minAHE	0.2766
	minFDE	1.4615
	Lane-changing accuracy	0.7936

**Table 6:** Trajectory and behavior prediction performance under different prediction horizons.

Setting	Dataset	ADERate	FDERate	minADE	minAHE	minFDE	minFHE	LaneChanging
3 s, 50 m	Validation	0.9756	0.9318	1.0496	0.2292	1.0247	0.1416	0.7927
	Test	0.9764	0.9301	1.0680	0.2299	0.8782	0.1449	0.7936
5 s, 70 m	Validation	0.9492	0.8830	1.2512	0.2568	1.1601	0.1688	0.7883
	Test	0.9479	0.8769	1.2707	0.2496	1.2241	0.1677	0.7902
6 s, 100 m	Validation	0.9231	0.8356	1.3707	0.2848	1.3930	0.1990	0.7890
	Test	0.9190	0.8320	1.3910	0.2766	1.4615	0.1891	0.7886

**Table 7:** Lane-changing behavior prediction accuracy under different prediction distances.

Prediction Distance (m)	20	30	40	50	70	100	200
Accuracy (%)	0.7950	0.7936	0.7923	0.7927	0.7883	0.7890	0.7910

## Data Availability

The data supporting the findings of this study are available from the authors upon reasonable request.

## Abbreviations

The following abbreviations are used in this manuscript:

EPSILON	Efficient Planning System for automated vehicles in highly interactive environments
DSAC	Distributional Soft Actor–Critic
ODD	Operational Design Domain
PODAR	Probabilistic Occupancy-based Dynamic Risk
HRI	Human–Robot Interaction
MR	Mobile Robot
QCNet	Query-Centric Network
RL	Reinforcement Learning
ADE	Average Displacement Error
FDE	Final Displacement Error
minADE	Minimum Average Displacement Error
minFDE	Minimum Final Displacement Error
minAHE	Minimum Average Heading Error
minFHE	Minimum Final Heading Error
MR	Miss Rate
